# Machine Learning–Based Prediction of Suicidality in Adolescents With Allergic Rhinitis: Derivation and Validation in 2 Independent Nationwide Cohorts

**DOI:** 10.2196/51473

**Published:** 2024-02-14

**Authors:** Hojae Lee, Joong Ki Cho, Jaeyu Park, Hyeri Lee, Guillaume Fond, Laurent Boyer, Hyeon Jin Kim, Seoyoung Park, Wonyoung Cho, Hayeon Lee, Jinseok Lee, Dong Keon Yon

**Affiliations:** 1 Department of Regulatory Science, Kyung Hee University Seoul Republic of Korea; 2 Center for Digital Health, Medical Science Research Institute, Kyung Hee University College of Medicine Seoul Republic of Korea; 3 Department of Pediatrics, Columbia University Irving Medical Center New York, NY United States; 4 Assistance Publique-Hôpitaux de Marseille, Research Centre on Health Services and Quality of Life, Aix Marseille University Marseille France; 5 Department of Biomedical Engineering, Kyung Hee University Yongin Republic of Korea; 6 Department of Electronics and Information Convergence Engineering, Kyung Hee University Yongin Republic of Korea; 7 Department of Pediatrics, Kyung Hee University College of Medicine Seoul Republic of Korea

**Keywords:** machine learning, allergic rhinitis, prediction, random forest, suicidality

## Abstract

**Background:**

Given the additional risk of suicide-related behaviors in adolescents with allergic rhinitis (AR), it is important to use the growing field of machine learning (ML) to evaluate this risk.

**Objective:**

This study aims to evaluate the validity and usefulness of an ML model for predicting suicide risk in patients with AR.

**Methods:**

We used data from 2 independent survey studies, Korea Youth Risk Behavior Web-based Survey (KYRBS; n=299,468) for the original data set and Korea National Health and Nutrition Examination Survey (KNHANES; n=833) for the external validation data set, to predict suicide risks of AR in adolescents aged 13 to 18 years, with 3.45% (10,341/299,468) and 1.4% (12/833) of the patients attempting suicide in the KYRBS and KNHANES studies, respectively. The outcome of interest was the suicide attempt risks. We selected various ML-based models with hyperparameter tuning in the discovery and performed an area under the receiver operating characteristic curve (AUROC) analysis in the train, test, and external validation data.

**Results:**

The study data set included 299,468 (KYRBS; original data set) and 833 (KNHANES; external validation data set) patients with AR recruited between 2005 and 2022. The best-performing ML model was the random forest model with a mean AUROC of 84.12% (95% CI 83.98%-84.27%) in the original data set. Applying this result to the external validation data set revealed the best performance among the models, with an AUROC of 89.87% (sensitivity 83.33%, specificity 82.58%, accuracy 82.59%, and balanced accuracy 82.96%). While looking at feature importance, the 5 most important features in predicting suicide attempts in adolescent patients with AR are depression, stress status, academic achievement, age, and alcohol consumption.

**Conclusions:**

This study emphasizes the potential of ML models in predicting suicide risks in patients with AR, encouraging further application of these models in other conditions to enhance adolescent health and decrease suicide rates.

## Introduction

### Background

Allergic rhinitis (AR) is a common atopic disorder that affects approximately 14% of the global population [1-3]. AR is called allergic rhinoconjunctivitis when the eyes are involved and is an inflammatory condition characterized by at least one of the following symptoms: nasal congestion, rhinorrhea, itching, and sneezing [4,5]. In addition, it has been found to affect quality of life measures such as sleep, physical and social functioning, and learning and memory [6]. Furthermore, AR has been found to be associated with depressive symptoms, including suicidal ideation and suicide attempts [7-13]. Suicide rates in adolescents continue to increase, and suicide is the leading cause of adolescent death in Korea [14]. Adolescents are by nature susceptible to mental health problems owing to the many transitions involved in this period of their life, including changes in school, living situations, pressures of fitting into peer groups, and building their own identity. This can invoke helplessness, insecurity, stress, and a loss of control, possibly accelerating suicide rates in adolescents, a leading cause of adolescent death in South Korea [14]. Owing to this substantially burden in adolescents, it is important to further understand and investigate potential methods of mitigating the risk that AR adds to an already pressing issue. There is a possibility that the suicide rate increased because of the decrease in the quality of life among adolescents with AR [15]. Therefore, we will predict the suicidal attempts among those with AR using machine learning (ML) models.

Suicide prediction is elusive and thus adds to the challenges of suicide prevention worldwide. No practical methods for anticipating individual suicides or stratifying individuals according to risk have been well established [16,17]; however, ML-based models is a potential method for more accurately identifying adolescents at risk of suicide. A systematic review on the prediction of self-injurious thoughts and behaviors with ML determined that despite its limited application, ML has made a significant advancement in suicide prediction [18]. Another review found that ML has the potential to improve suicide predictions compared with traditional suicide prediction models [18]. Such studies illustrate the rapidly growing potential of ML.

### Objectives

Given the additional risk of suicide-related behaviors in adolescents with AR, it would be relevant to use the growing field of ML to better evaluate this risk. Using nationwide population data, this study aimed to develop an ML-based model to predict suicide attempts among patients with AR using 2 independent nationwide cohorts in South Korea. We expect that this ML model produced from these data will have a high balanced accuracy and area under the receiver operating characteristic curve (AUROC) and consequently assist in better understanding suicide risk in adolescents with AR.

## Methods

### Study Design and Participants

**Figure 1:**
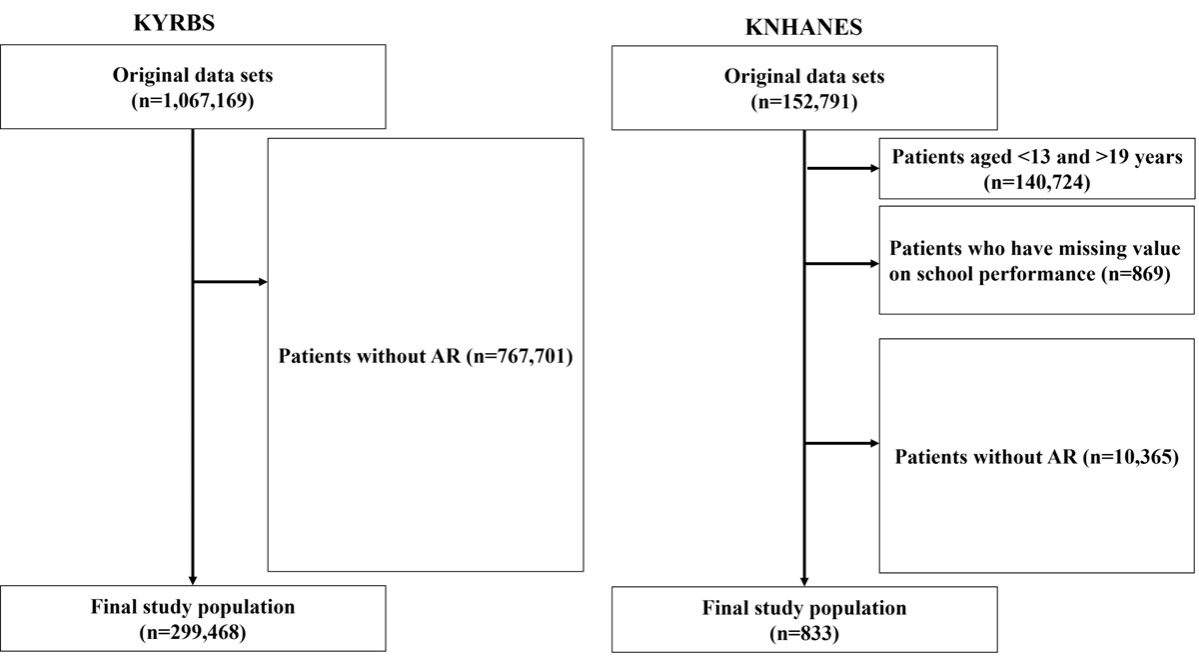
Study workflow. AR: allergic rhinitis; KNHANES: Korea National Health and Nutrition Examination Survey; KYRBS: Korea Youth Risk Behavior Web-based Survey.

This study aimed to develop an ML model to predict suicidality in Korean adolescents aged 13 to 18 years using clinical features extracted from 2 large independent data sets: the Korea Youth Risk Behavior Web-based Survey (KYRBS) and the Korea National Health and Nutrition Examination Survey (KNHANES) [19,20]. Figure 1 shows the workflow diagrams of the KYRBS and KNHANES data sets, which both offer nationally representative samples and estimates of the total adolescent population in South Korea. The original sample size for KYRBS was 1,067,169. However, after excluding patients without totaling 767,701, the final study population in the KYRBS data set was reduced to 299,468. Similarly, the KNHANES data set initially comprised 152,791 participants. However, after excluding 140,724 individuals either aged <13 or >19 years, 869 individuals with missing values on school performance, and 10,365 individuals without AR, the final study population in the KNHANES data set was 833.

We included Korean adolescents aged between 13 and 18 years who completed the survey between 2005 and 2021 in KYRBS and between 1998 and 2021 in KNHANES. The outcome of suicidality was defined for people who attempted suicide more than once within 1 year [12], and covariates included age, sex, BMI (kg/m^2^), residential area, household income, parents’ level of education, academic achievement, smoking status, stress status, and feelings of sadness and despair.

We trained, validated, and externally tested the ML model’s predictive accuracy and potential clinical efficacy in identifying the presence of mental health conditions using data from adolescents who met the same inclusion and exclusion criteria as those in the KYRBS data set. This study followed the guidelines outlined in the Transparent Reporting of a Multivariable Prediction Model for Individual Prognosis or Diagnosis statement (Table S1 in Multimedia Appendix 1).

### Ethical Considerations

The study protocol was approved by the institutional review board of the Korean Centers for Disease Control and Prevention Agency and Kyung Hee University (2022-06-042), and all participants provided written informed consent.

### Variables and Algorithm Selection

To solve the imbalance issue of our data set, we used the synthetic minority oversampling technique to balance the training data set. The synthetic minority oversampling technique synthesizes new data from existing data using k-nearest neighbors and inserts them into the original data set [21]. The data set was randomly divided into 4:1 at a base training set (462,203/577,854, 79.98%) and a base test set (115,651/577,854, 20.01%) with equal distribution of different classes of patient data. This study aimed to develop a predictive model with a small number of variables and good performance; a model trained with the basic training set is needed to compare with a model with fewer variables than the basic training set. In addition, a corresponding test set was required to evaluate each training set, including the basic training set. Continuous variables were compared using the 2-tailed *t* test or Mann-Whitney *U* test, and categorical variables were compared using the chi-square test [22]. The odds ratios of the variables were determined by logistic regression (method: enter). Data set variables were analyzed using SAS software (version 9.3; SAS Institute Inc).

### ML Model

**Figure 2:**
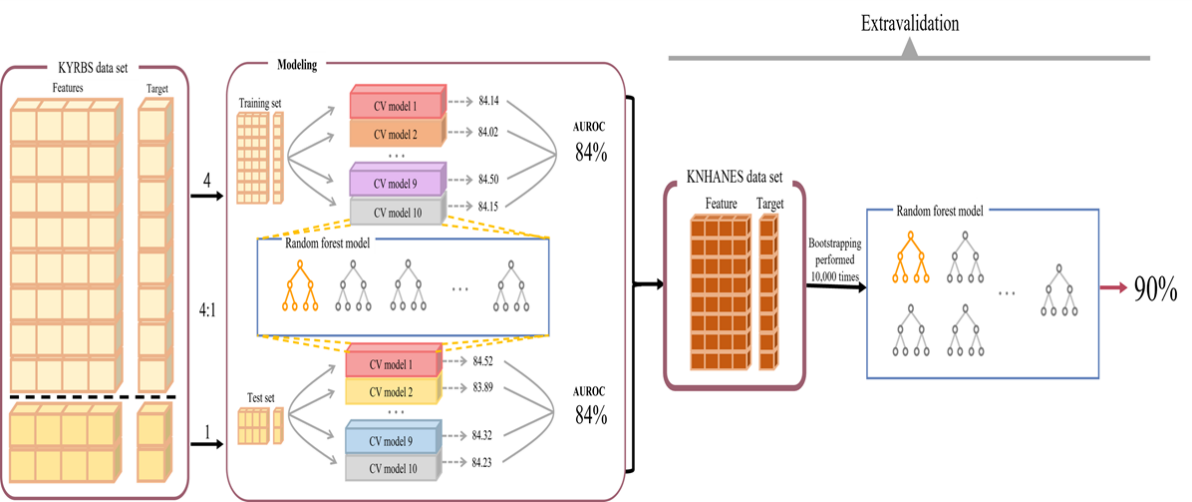
Model architecture. The original Korea Youth Risk Behavior Web-based Survey (KYRBS) data set was partitioned into training and test sets in a 4:1 ratio. The training set was used for model development, with performance assessed using area under the receiver operating characteristic curve (AUROC) scores on the test set. Selected high-performing models were further validated using an external Korea National Health and Nutrition Examination Survey (KNHANES) data set. The train data results were derived from the training data set, the test results were derived from the test data set, and the external results were derived from the additional KNHANES data set. CV: cross validation.

In this study, as depicted in Figure 2, we analyzed the original KYRBS data set. The data set was divided into training and test data sets using a 4:1 ratio, with the training data set being used for model development and the test data set being used for model evaluation. We applied various ML algorithms to the training data set and assessed their performance based on the AUROC scores on the test data set. Models that exhibited high performance were selected for further investigation.

For external validation, we used an additional data set from the KNHANES, which contained the same columns as the original KYRBS data set. To enhance our understanding of the model’s performance and its variability, we applied bootstrapping techniques. Bootstrapping was repeated 10,000 times to evaluate the model’s performance on the external data set [23]. This process involved creating numerous resamples from the data set, with each sample being used to calculate the model’s performance metrics. We calculated the mean and SE of these performance metrics across all the bootstrap samples. This technical approach provided a robust measure of the model’s performance, accounting for variability and uncertainty in the external data set. The performance of the selected models on this external data set was evaluated, and their performance metrics were compared with those obtained from the training and test data sets. In summary, the train results were derived from the training data set, the test results were derived from the test data set, and the external results were derived from the external validation data set provided by KNHANES.

As shown in Figure 1, in the data preprocessing phase of this study, we took several steps to clean and prepare the data for efficient analysis. We used SAS software for data processing, which included categorizing and handling the missing values from the data set from the survey. Moreover, to address variables present in KYRBS but absent in KNHANES, we filled the missing values in KNHANES with the median values from KYRBS. These steps included handling missing values, categorizing categorical variables, and scaling numerical features using SAS software. The preprocessing steps aimed to ensure that the data were in a suitable format for the subsequent application of various ML algorithms. It is crucial to preprocess the data effectively, as this can substantially impact the performance and generalizability of the models being developed. Moreover, we used a 10-fold cross-validation approach to assess the performance of the ML models more reliably. This method involves partitioning the original data set into 10 equal-sized subsets, with each subset being used as a test data set once, whereas the remaining subsets serve as the training data set. The process was repeated 10 times, and the performance metrics, such as the AUROC score, sensitivity, specificity, accuracy, balanced accuracy score, precision, and *F*_1_-score, were averaged over these iterations.

To estimate the uncertainty and variability of our results, we calculated the 95% CIs for each performance metric, including the AUROC score, sensitivity, specificity, accuracy, balanced accuracy score, precision, and *F*_1_-score during the 10-fold cross-validation process to the train and test data sets. The 95% CI provides a range of plausible values for the performance metrics and is a useful tool for determining the stability and generalizability of the models. Data processing was performed using SAS software, and ML analysis was performed using Python (version 3.9.16), TensorFlow-gpu (version 2.6.0), Keras (version 2.6.0), NumPy (version 1.23.5), *pandas* (version 1.5.3), *scikit-learn* (version 1.2.2), Matplotlib (version 3.7.1), and *shap* (version 0.42.1). The ML models that were used were tree-based models, which are random forest, XGBoost, AdaBoost, and light gradient boost. We used GridSearch to fine-tune the hyperparameters of the models with the objective of maximizing the AUROC scores. The model hyperparameters were tuned using GridSearch, which uses many combinations of different hyperparameters to obtain the best AUROC score. GridSearch is an exhaustive search method that systematically explores a range of hyperparameter combinations, evaluating the performance of each combination on the given data set. By selecting the optimal set of hyperparameters from multiple variables using GridSearch, we selected from a range of hyperparameters for the random forest model. Finally, we chose number estimators at 100, maximum depth at 6, and maximum features as *sqrt*. This approach aimed to improve the performance and generalizability of our models, ultimately leading to more accurate and reliable predictions.

Subsequently, we focused on a detailed analysis of feature impact within the model. Feature importance was assessed using the mean decrease in impurity within the random forest model, indicating how each feature contributes to more uniform node splits. In addition, the Seaborn library was used for visualizing this importance, enhancing the interpretability of the results, and aiding in the identification of the most impactful features for the model’s predictive accuracy. This approach provides effective feature selection and model optimization.

To interpret and gain insights into the model’s predictions, we calculated the Shapley Additive Explanations (SHAP) values from the random forest model. SHAP is a popular model-agnostic, local explanation approach designed to explain any given classifier. Lundberg and Lee [24] proposed the SHAP value as a united approach to explain the output of any ML model. We used the force plot and waterfall plot of the random forest model. This visualizes the contribution of each feature to the model’s prediction for a specific instance, showing how each feature pushes the model’s output from the base value. In contrast, the waterfall plot provides a detailed, step-by-step breakdown of how each feature contributes to moving the model’s output from the expected value to the actual prediction.

### Software and Libraries

All computations, model training, and evaluations were executed using Python (version 3.9.16), TensorFlow-gpu (version 2.6.0), Keras (version 2.6.0), NumPy (version 1.23.5), *pandas* (version 1.5.3), *scikit-learn* (version 1.2.2), and Matplotlib (version 3.7.12) for ML tasks and data wrangling. Visualization was facilitated using Matplotlib (version 3.7.2), Seaborn (version 0.12.2), and *shap* (version 0.42.1).

## Results

### Demographic Characteristics

This study was conducted using nationwide population data from 2 independent cohorts in South Korea to develop and investigate an ML-based model for predicting suicide attempts in patients with AR. The demographic characteristics of the study population were as follows: both cohorts consisted of patients with AR, with the KYRBS cohort including 299,468 patients and the KNHANES cohort comprising 833 adolescents aged 13 to 18 years. Table 1 shows the baseline characteristics of the KYRBS and KNHANES. In the original KYRBS cohort, the sex distribution revealed that among 299,468 patients, 152,789 (51.02%) were male patients and 146,679 (48.98%) were female patients. In the extravalidated KNHANES cohort, comprising 833 patients, 492 (59.06%) were male patients and 341 (40.94%) were female patients. The patient samples in both cohorts encompassed diverse socioeconomic backgrounds, including varying levels of education, income, and occupation. Overall, the study included a total of 300,301 patients with AR from diverse demographic backgrounds, ensuring a representative sample for the development and evaluation of the ML model. By considering these demographic characteristics, this study aimed to provide valuable insights into the risk of suicide attempts among individuals with AR, with a particular focus on the adolescent population.

**Table 1:** Demographic characteristics of Korea Youth Risk Behavior Web-based Survey (KYRBS) data (2005-2022) and Korea National Health and Nutrition Examination Survey (KNHANES) data cohorts (1998-2022) among excluded and included participants.

Characteristics		KYRBS				KNHANES		
		Total	AR^a^	Non-AR	Total		AR	Non-AR
Total, n		1,067,169	299,468	767,701	6306		833	5473
Age (years), mean (SD)		15.41 (1.69)	15.55 (1.69)	15.35 (1.69)	15.26 (1.65)		15.12 (1.65)	15.29 (1.65)
**Sex, n (%)**								
	Male	548,952 (51.44)	152,789 (51.02)	396,134 (51.60)	3338 (52.93)		492 (59.06)	2846 (52.00)
	Female	518,217 (48.56)	146,679 (48.98)	371,567 (48.40)	2968 (47.07)		341 (40.94)	2627 (48.00)
**Region, n (%)**								
	Urban	495,593 (46.44)	141,229 (47.16)	354,418 (46.17)	5172 (82.02)		716 (85.95)	4456 (81.42)
	Rural	571,576 (53.56)	158,239 (52.84)	413,283 (53.84)	1134 (17.98)		117 (14.05)	1017 (18.58)
**BMI (kg/m^2^), n (%)^b^**								
	Underweight (<18.5)	265,832 (24.91)	69,716 (23.28)	196,148 (25.55)	1392 (22.07)		166 (19.93)	1226 (22.40)
	Normal (18.5-23.0)	565,813 (53.02)	159,736 (53.34)	406,036 (52.89)	3199 (50.73)		396 (47.54)	2803 (51.22)
	Overweight (23.0-25.0)	120,270 (11.27)	35,368 (11.81)	84,908 (11.06)	755 (11.97)		112 (13.45)	643 (11.75)
	Obese (≥25.0)	115,254 (10.80)	34,678 (11.58)	80,609 (10.50)	960 (15.22)		159 (19.09)	801 (14.64)
**Smoking status, n (%)**								
	Nonsmoker	850,534 (79.70)	244,576 (81.67)	605,870 (78.92)	5945 (94.28)		794 (95.32)	5151 (94.12)
	Smoker	216,635 (20.30)	54,892 (18.33)	161,831 (21.08)	361 (5.72)		39 (4.68)	322 (5.88)
**Alcohol consumption, n (%)**								
	Nondrinker	868,996 (81.43)	247,540 (82.66)	621,376 (80.94)	4965 (78.73)		678 (81.39)	4287 (78.33)
	1-2 days	115,361 (10.81)	30,995 (10.35)	84,294 (10.98)	1009 (16.00)		126 (15.13)	883 (16.13)
	3-5 days	36,284 (3.40)	9643 (3.22)	26,716 (3.48)	239 (3.79)		23 (2.76)	216 (3.95)
	6-9 days	21,770 (2.04)	5301 (1.77)	16,506 (2.15)	57 (0.90)		6 (0.72)	51 (0.93)
	≥10 days	24,758 (2.32)	5989 (2.00)	18,809 (2.45)	36 (0.57)		0 (0.00)	36 (0.66)
**Parents’ highest educational level, n (%)**								
	University graduate or higher	485,455 (45.49)	160,545 (53.61)	324,841 (42.32)	N/A^c^		N/A	N/A
	High school graduate	283,440 (26.56)	67,919 (22.68)	215,467 (28.07)	N/A		N/A	N/A
	Middle school graduate or below	19,209 (1.80)	2995 (1.00)	16,275 (2.12)	N/A		N/A	N/A
	Unknown	279,065 (26.15)	68,009 (22.71)	211,118 (27.50)	N/A		N/A	N/A
**Academic achievement (percentile), n (%)**								
	Low (0-19)	99,674 (9.34)	24,586 (8.21)	75,081 (9.78)	N/A		N/A	N/A
	Lower middle (20-39)	248,117 (23.25)	64,535 (21.55)	183,511 (23.91)	N/A		N/A	N/A
	Middle (40-59)	310,226 (29.07)	83,822 (27.99)	226,442 (29.50)	N/A		N/A	N/A
	Upper middle (60-79)	276,397 (25.90)	83,162 (27.77)	193,307 (25.18)	N/A		N/A	N/A
	High (80-100)	132,755 (12.44)	43,363 (14.48)	89,360 (11.64)	N/A		N/A	N/A
**Household income, n (%)**								
	Low (0-39)	163,277 (15.30)	43,962 (14.68)	119,301 (15.54)	805 (12.77)		71 (8.52)	734 (13.41)
	Middle (40-59)	523,767 (49.08)	143,745 (48.00)	380,012 (49.50)	1573 (24.94)		198 (23.77)	1375 (25.12)
	Upper middle (60-79)	292,298 (27.39)	85,977 (28.71)	206,281 (26.87)	2034 (32.26)		282 (33.85)	1752 (32.01)
	High (80-100)	87,828 (8.23)	25,784 (8.61)	62,107 (8.09)	1894 (30.03)		282 (33.85)	1612 (29.45)
**Asthma, n (%)**								
	No	990,013 (92.77)	255,596 (85.35)	73,4383 (95.66)	6095 (96.65)		763 (91.60)	5332 (97.42)
	Yes	77,156 (7.23)	43,872 (14.65)	33,318 (4.34)	211 (3.35)		70 (8.40)	141 (2.58)
**Dermatitis, n (%)**								
	No	856,083 (80.22)	203,758 (68.04)	652,316 (84.97)	5666 (89.85)		639 (76.71)	5027 (91.85)
	Yes	211,086 (19.78)	95,710 (31.96)	115,385 (15.03)	640 (10.15)		194 (23.29)	446 (8.15)
**Stress status, n (%)^d^**								
	Mild	189,743 (17.78)	47,166 (15.75)	142,562 (18.57)	956 (15.16)		105 (12.61)	851 (15.55)
	Moderate	449,705 (42.14)	123,860 (41.36)	325,862 (42.44)	3629 (57.55)		458 (54.98)	3171 (57.94)
	High	312,467 (29.28)	92,745 (30.97)	219,743 (28.62)	1465 (23.23)		237 (28.45)	1228 (22.44)
	Severe	115,254 (10.80)	35,697 (11.92)	79,534 (10.36)	256 (4.06)		33 (3.96)	223 (4.07)
**Sadness and despair in the past year, n (%)**								
	No	740,615 (69.40)	202,830 (67.73)	537,775 (70.05)	5861 (92.94)		764 (91.72)	5097 (93.13)
	Yes	326,554 (30.60)	96,638 (32.27)	229,926 (29.95)	445 (7.06)		69 (8.28)	376 (6.87)
**Suicidal thoughts in the past year, n (%)**								
	No	898,023 (84.15)	250,265 (83.57)	647,709 (84.37)	5509 (87.36)		788 (94.60)	4721 (86.26)
	Yes	169,146 (15.85)	49,203 (16.43)	119,992 (15.63)	797 (12.64)		45 (5.40)	752 (13.74)
**Suicide attempt in the past year, n (%)**								
	No	1,030,779 (96.59)	289,136 (96.55)	741,599 (96.60)	6241 (98.97)		821 (98.56)	5420 (99.03)
	Yes	36,390 (3.41)	10,332 (3.45)	26,102 (3.40)	65 (1.03)		12 (1.44)	53 (0.97)

^a^AR: allergic rhinitis.

^b^According to Asia-Pacific guidelines, BMI is divided into 4 groups: underweight (<18.5 kg/m^2^), normal (18.5-22.9 kg/m^2^), overweight (23.0-24.9 kg/m^2^), and obese (≥25.0 kg/m^2^).

^c^N/A: not applicable.

^d^Stress was defined by receipt of mental health counseling owing to stress.

### ML Model Results

**Figure 3:**
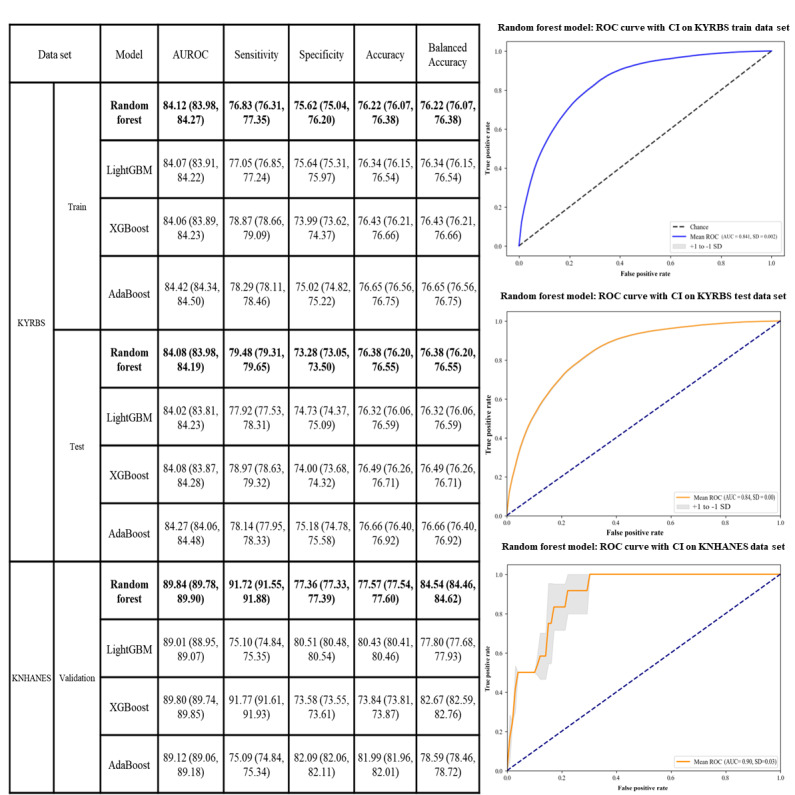
The area under the receiver operating characteristic curve (AUROC) of 4 different machine learning algorithms in the train, test, and external validation data set. KNHANES: Korea National Health and Nutrition Examination Survey; KYRBS: Korea Youth Risk Behavior Web-based Survey; ROC: receiver operating characteristic.

As shown in Figure 3 and Figure S1 in Multimedia Appendix 1, upon conducting extensive model evaluations, it was found that the random forest model was the best model in predicting suicide attempts in patients with AR. The train data results revealed that the random forest model achieved a sensitivity of 76.83 (95% CI 76.31-77.35), a specificity of 75.62 (95% CI 75.04-76.20), an accuracy of 76.22 (95% CI 76.07-76.38), a balanced accuracy of 76.22 (95% CI 76.07-76.38), a precision of 75.91 (95% CI 75.57-76.25), an *F*_1_-score of 76.37 (95% CI 76.19-76.54), and an AUROC of 84.12 (95% CI 83.98-84.27).

In contrast, the AdaBoost model yielded slightly different results with a sensitivity of 78.29 (95% CI 78.11-78.46), a specificity of 75.02 (95% CI 74.82-75.22), an accuracy of 76.65 (95% CI 76.56-76.75), a precision of 75.81 (95% CI 75.68-75.94), an *F*_1_-score of 77.03 (95% CI 76.93-77.12), and an AUROC of 84.42 (95% CI 84.34-84.50).

However, when these models were evaluated on a separate test set, their performance varied. The random forest model obtained a sensitivity of 77.61 (95% CI 77.43-77.79), a specificity of 75.03 (95% CI 74.83-75.23), an accuracy of 76.32 (95% CI 76.16-76.49), a balanced accuracy of 76.32 (95% CI 76.16-76.49), a precision of 75.66 (95% CI 75.49-75.83), an *F*_1_-score of 76.62 (95% CI 76.46-76.78), and an AUROC of 84.18 (95% CI 84.07-84.28). Conversely, the AdaBoost model showed a sensitivity of 78.14 (95% CI 77.95-78.33), a specificity of 75.18 (95% CI 74.78-75.58), an accuracy of 76.66 (95% CI 76.40-76.92), a balanced accuracy of 76.66 (95% CI 76.40-76.92), a precision of 75.89 (95% CI 75.57-76.21), an *F*_1_-score of 77.00 (95% CI 76.77-77.23), and an AUROC of 84.27 (95% CI 84.06-84.48).

For external validation, an independent data set, KNHANES, was used. The random forest model achieved a sensitivity of 91.72 (95% CI 91.55-91.88), a specificity of 77.36 (95% CI 77.33-77.39), an accuracy of 77.57 (95% CI 77.54-77.60), a balanced accuracy of 84.54 (95% CI 84.46-84.62), and an AUROC of 89.84 (95% CI 89.78-89.90). Meanwhile, the AdaBoost model’s external validation results revealed a sensitivity of 75.09 (95% CI 74.84-75.34), a specificity of 82.09 (95% CI 82.06-82.11), an accuracy of 81.99 (95% CI 81.96-82.01), a balanced accuracy of 78.59 (95% CI 78.46-78.72), and an AUROC of 89.12 (95% CI 89.06-89.18).

On the basis of the comprehensive results, the random forest model demonstrated superior performance compared with the AdaBoost model when evaluated on both internal and external data sets. In addition, the area under the precision-recall curve for the random forest model, a measure of model performance under conditions of class imbalance, was 81.98 (95% CI 79.88-84.08), as shown in Figure S1 in Multimedia Appendix 1. This indicates the model’s robust ability to maintain precision across various levels of recall.

### Feature Importance

Table 2 shows that the random forest model identified sadness and despair (53.1%) as the most influential feature in predicting suicide attempts in patients with AR, followed by stress status (28.35%), academic achievement (5.18%), age (4.08%), alcohol consumption (2.96%), household income (1.65%), sex (1.56%), smoking status (1.33%), BMI (kg/m^2^; 0.69%), region (0.47%), parents’ highest educational level (0.27%), atopic dermatitis (0.23%), and asthma (0.14%) in descending order of importance.

**Table 2:** Feature importance of the random forest model.

Feature	Importance, %
Sadness and despair	53.1
Stress status	28.35
Academic achievement	5.18
Age	4.08
Alcohol consumption	2.96
Household income	1.65
Sex	1.56
Smoking status	1.33
BMI (kg/m^2^)^a^	0.69
Region	0.47
Parents' highest educational level	0.27
Atopic dermatitis	0.23
Asthma	0.14

^a^According to Asia-Pacific guidelines, BMI is divided into 4 groups: underweight (<18.5 kg/m^2^), normal (18.5-22.9 kg/m^2^), overweight (23.0-24.9 kg/m^2^), and obese (≥25.0 kg/m^2^).

### SHAP Value

We addressed a deeper visual interpretation of the SHAP values within our ML model. Figure S2 in Multimedia Appendix 1 shows a waterfall plot, distinctively showcasing the cumulative contribution of each feature to a single prediction. We interpreted individual predictions by starting from the initial estimate and sequentially incorporating the influence of each feature to reach the final prediction. E[f(x)] refers to the average predicted output of the model across the entire data set, providing insights into the model’s overall prediction tendency. The starting point of the illustration, denoted as E[f(X)]=0.50, represents the model’s average prediction for the given data set. Among the variables, sadness and despair stood out, boosting the prediction by 0.16 and ranking as the most influential factor. Conversely, stress status, school performance, and sex reduced the prediction by 0.1, 0.02, and 0.01, respectively. This visualization offers a clear insight into the profound influence each feature wields in predicting adolescent suicidal thinking. Our ML model notably underscores substantial reliance on sadness and despair and stress status features. Moreover, in the force plot, features pushing the prediction higher are usually shown in one red color, whereas those pushing the prediction lower are shown in blue, clearly displaying the push and pull effect of each feature on the model’s prediction. This type of visualization will allow us to see the balance of each effect at each individual prediction level, further clarifying the roles of sadness despair, stress status, and other features in assessing the risk of adolescent suicide attempts.

### Code Availability

On the basis of the results of the ML model, we established a web-based application for policy makers or health system managers to support their decision-making process for cases involving suicidal attempts in adolescents with AR [25]. An example of a web interface and the results is shown in Figure S3 in Multimedia Appendix 1. Custom code for the website is available on the internet [26].

## Discussion

### Principal Findings

The study results showed that ML models can predict suicide attempts in patients with AR with relatively high accuracy. The random forest model is the best ML model to predict suicide attempts among Korean adolescents with AR, with an AUROC of 84.12% (original data set) and 89.87% (external validation data set). While looking at feature importance, the 5 most important features in predicting suicide attempts in adolescent patients with AR are depression, stress status, academic achievement, age, and alcohol consumption.

To our knowledge, this is the first study to use an ML model in the context of patients with AR and suicidality, especially at this population level. These results reinforce the importance for clinicians to pay close attention to atopic conditions such as AR when screening for suicide risk as well as to help identify the most important risk factors in such patients.

### Comparison With Previous Studies

In concordance with our findings, studies on suicide-related behavior prediction using ML have shown great promise. A study found that ML for suicide risk prediction in children and adolescents with electronic health records was able to detect 53% to 62% of suicide-positive participants with 90% specificity [27], and a case-control study of first-time suicide attempts with a cohort of >45,000 patients demonstrated accurate and robust first-time suicide attempt prediction [28], with the best predicting model achieving an AUROC of 0.932. A study that used the Korea Welfare Panel Study to develop an ML algorithm determined that >80% of individuals at risk of suicide-related behaviors could be predicted by various mental and socioeconomic characteristics of the respondents [29]. In addition, ML together with in-person screening has been found to result in the best suicide risk prediction [30], illustrating its potential to be used by clinicians in the medical field. These studies, as well as our study, support the continued need to build and improve ML models for predicting suicide risk, especially for at-risk patients.

As discussed, adolescents remain at a high risk for suicide-related behaviors because of their unique social situation. A study identified that significant risk factors for suicide in youth include a history of mental disorders, previous suicide attempts, impulsivity, family structure or environment, interpersonal strain, school problems and academic stress status, etc [31]. This is reflected in the findings of this study that showed adolescent’s age, academic achievement, BMI group, and household income as some of the most important contributors to suicide attempt prediction in adolescents with AR. In addition, it has been observed that atopic dermatitis and asthma, although less common than the other risk factors, contribute to this already high burden of risk.

### Plausible Mechanism

This study shows the importance of understanding what puts adolescents at risk for suicide, especially in the context of AR. There is a proposed pathogenic mechanism that connects atopy and its associated risk of increased suicidality. Allergic inflammatory mediators, interleukin (IL)-4, IL-5, and IL-13, are released and perpetuated by “allergic” helper T subtype 2 (T_H_2) cells [32,33]. Along with atopic dermatitis and asthma, AR is associated with systemic increases in such cytokines [34]. Early life overexposure to IL-4, which can occur because of T_H_2 sensitization from allergic disease, has been reported to reduce myelination and lead to cognitive impairment and developmental delays [35], and these effects have been found to be inhibited with IL-4 neutralization [36]. Allergy-mediated cytokines can also lead to aberrations in rapid eye movement (REM) sleep, increased REM latency, increased arousal, and decreased REM duration [37], thereby reducing sleep quality, quality of life, and overall happiness. In addition, T_H_2 sensitization has been shown to possibly lead to negative effects on the developing brain, leading to increased attention-deficit/hyperactivity disorder, depression, anxiety, and suicidal ideation [38]. These mechanisms of action point toward a functional correlation between atopy and psychological disorders, including depression. This study helps to elucidate which other risk factors further contribute to such patients’ already increased risk.

### Strengths and Limitations

This study had several limitations. As the data sets only contained Korean adolescents, this model may not extend to the global adolescent population. South Korea’s unique cultural and environmental setting may particularly affect the generalizability of the study. In the validation data set, participants aged <13 and >19 years were treated as missing data, and only patients with AR were analyzed, resulting in a low figure of 1%. This is because the KNHANES data set targets all ages, and not just adolescents; hence, it lacks the specificity for adolescents compared with the KYRBS data set. However, this data set represents South Korea and is used in studies as an external validation for the KYRBS data set [39-41]. In addition, the risk calculator that we produced was purely created for academic purposes, and its application should be limited to that scope. It is to be used as an example of what could be developed in the future with further refinement of ML models and our understanding of AR and suicide risk. The observed results are not intended to guide clinical management at this stage. As for the strengths of our study, to the best of our knowledge, this is the first study to create an ML model to predict suicide attempts in adolescents with AR. It is important to continue to investigate the role of atopic conditions in exacerbating suicide risk and to further understand how such patients may be affected by their disease process in the context of depression and suicide-related behaviors. Our model has the potential to make a significant impact on improving suicide risk assessment, early identification, and effective interventions for patients with AR, and it is important to further investigate the usefulness of ML and suicidality in patients with atopic diseases.

### Clinical and Policy Implications

The findings of this study have various implications for clinicians and policy makers. It reveals the importance of screening adolescent patients with allergic diseases such as AR for suicidality. It can be assumed that as symptoms of atopy worsen, the risk for suicide also increases, and thus, it is important to encourage physicians to treat and pay close attention to their patients with allergies. These findings may also encourage psychiatrists to begin screening for allergies in their patients with depression or who are at risk of suicide attempts. Moreover, this ML-derived algorithm can be used by clinicians to independently screen their patients for the risk of suicide attempts. This can be done quickly and efficiently in the outpatient setting and can be incorporated as a tool to improve health outcomes for patients with atopic diseases. The relatively high accuracy of this model encourages further research into developing similar ML models for other atopic diseases, including atopic dermatitis and asthma. For policy makers, these findings illustrate the importance of raising awareness of the contribution of allergic diseases, especially untreated ones, to increased rates of suicide. As part of this awareness, it is critical to highlight the potential benefits of interventions such as anti-inflammatory diets and fasting on mental health [42,43]. If the general population understands the risks, they can become more diligent in bringing adolescent patients to their clinicians to be screened and treated for their allergies. They will then be able to use an ML model such as this study to uniquely understand each patient’s individualized suicide risk based on their various risk factors, including academic achievement and stress status. From this perspective, this ML algorithm holds great potential for improving the lives of adolescents affected by AR and its consequences.

### Conclusions

ML models are a new and innovative field of study that show great potential in predicting suicide, a task that has proven difficult for clinicians thus far. This study confirms this potential and demonstrates its accuracy in the context of AR. Being able to identify patients with a specific risk factor and understand their unique risks for suicide is incredibly important and relevant for clinicians of all different specialties. This encourages the development of ML models not only in other atopic conditions such as asthma and atopic dermatitis but also in conditions outside of atopy. Further research should be conducted to investigate the utility of these ML models in the clinical field with the goal of decreasing suicide rates in the already vulnerable group that is adolescents. Although much research remains to be done, this new and exciting field of ML holds promise for improving the health of patients with atopic diseases.

## Appendix

Multimedia Appendix 1 Supplement material and Transparent Reporting of a Multivariable Prediction Model for Individual Prognosis or Diagnosis statement.

## Abbreviations

AR: allergic rhinitis
AUROC: area under the receiver operating characteristic curve
IL: interleukin
KNHANES: Korea National Health and Nutrition Examination Survey
KYRBS: Korea Youth Risk Behavior Web-based Survey
ML: machine learning
REM: rapid eye movement
SHAP: Shapley Additive Explanations
TH2: helper T subtype 2

## Data Availability

Data are available upon reasonable request. Study protocol and statistical code are available from DKY. The data set is available from the Korea Disease Control and Prevention Agency through a data use agreement.
