# Supplementary material for: Machine Learning–Based Prediction of Suicidality in Adolescents With Allergic Rhinitis: Derivation and Validation in 2 Independent Nationwide Cohorts
Source: J Med Internet Res. 2024 Feb 14;26:e51473. doi: 10.2196/51473 (PMC10902766; doi:10.2196/51473)
Supplement: Multimedia Appendix 1 [file jmir_v26i1e51473_app1.docx]

| **Supplement Material** |
| --- |

Original article

**Machine learning-based prediction of suicidality in adolescents with allergic rhinitis: Derivation and validation in two independent nationwide cohorts in South Korea**

**Running title:** Suicidality and allergic rhinitis

Hojae Lee^1,2∥^, Joong Ki Cho^3∥^, Jaeyu Park^1,2∥^, Hyeri Lee^1,2∥^, Guillaume Fond^4^, Laurent Boyer^4^, Hyeon Jin Kim^1,2^, Seoyoung Park^5^, Wonyoung Cho^1^, Hayeon Lee^1,5*^, Jinseok Lee^5,6*^, Dong Keon Yon^1,2,7*^

1. Center for Digital Health, Medical Science Research Institute, Kyung Hee University Medical Center, Kyung Hee University College of Medicine, Seoul, South Korea

2. Department of Regulatory Science, Kyung Hee University, Seoul, South Korea

3. Department of Pediatrics, Columbia University Irving Medical Center, New York, NY, USA

4. Research Centre on Health Services and Quality of Life, Aix Marseille University, Marseille, France

5. Department of Biomedical Engineering, Kyung Hee University, Yongin, South Korea

6. Department of Electronics and Information Convergence Engineering, Kyung Hee University, Yongin, South Korea

7. Department of Pediatrics, Kyung Hee University Medical Center, Kyung Hee University College of Medicine, Seoul, South Korea

^∥^ These authors contributed equally.

***Corresponding authors**

**Figure S1.** Precision and F1-score of four different machine learning algorithms in the train and test dataset of KYRBS among AR patients and area under the precision recall curve on random forest model. AUPRC, area under the precision recall curve; CI, confidence interval; KYRBS, Korea Youth Risk Behavior Web-based Survey.

**
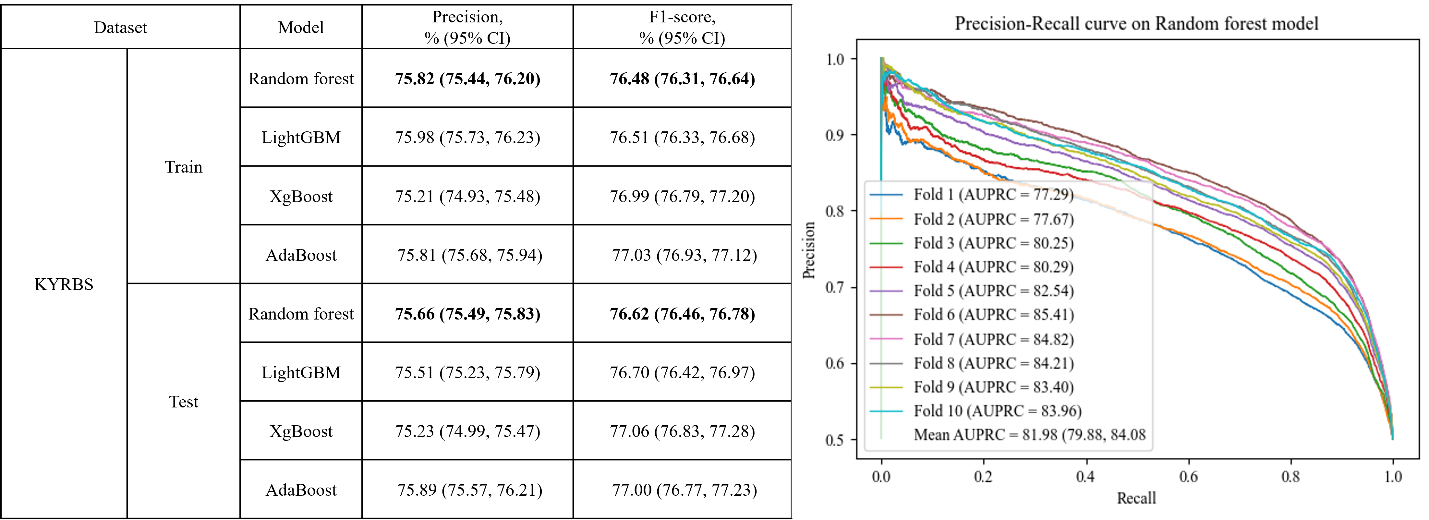
**

**Figure S2**. SHAP value on random forest model


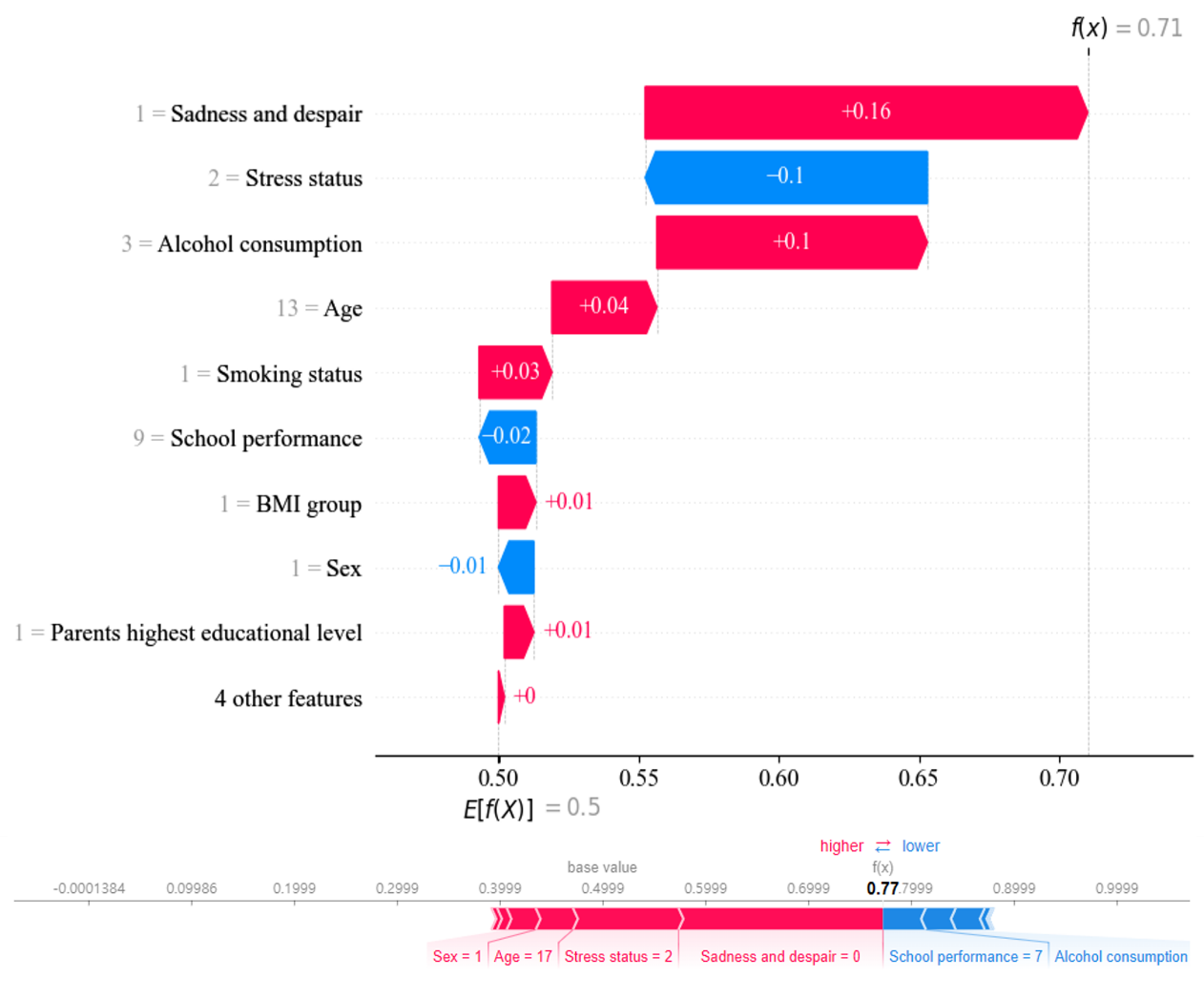


**Figure S3.** Deployed web-based application to provide suicidal attempts prediction among adolescents: a user's web interface to enter information and the prediction results with the probability of suicidal attempts.


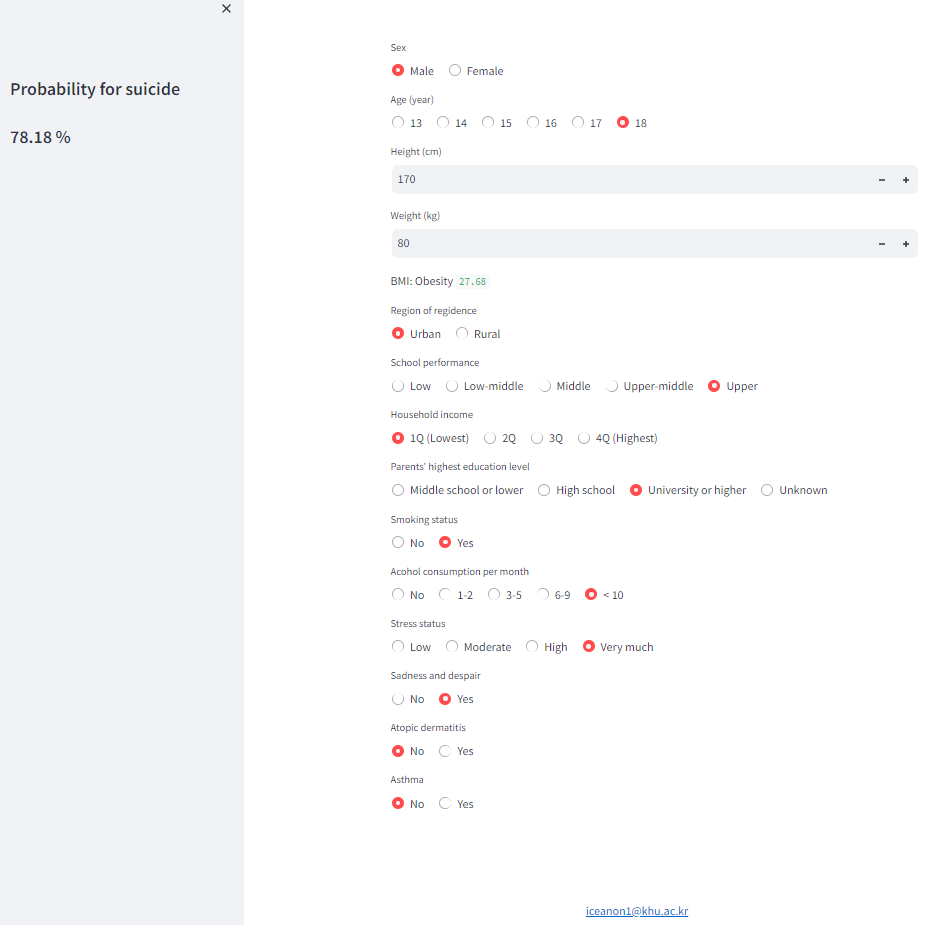


| **Section/Topic** | **Item** |  | **Checklist Item** | **Page** |
| --- | --- | --- | --- | --- |
| **Title and abstract** | | | | |
| Title | 1 | D;V | Identify the study as developing and/or validating a multivariable prediction model, the target population, and the outcome to be predicted. | 1 |
| Abstract | 2 | D;V | Provide a summary of objectives, study design, setting, participants, sample size, predictors, outcome, statistical analysis, results, and conclusions. | 3 |
| **Introduction** | | | | |
| Background and objectives | 3a | D;V | Explain the medical context (including whether diagnostic or prognostic) and rationale for developing or validating the multivariable prediction model, including references to existing models. | 3-5 |
|  | 3b | D;V | Specify the objectives, including whether the study describes the development or validation of the model or both. | 5-6 |
| **Methods** | | | | |
| Source of data | 4a | D;V | Describe the study design or source of data (e.g., randomized trial, cohort, or registry data), separately for the development and validation data sets, if applicable. | 7  Figure 1 |
|  | 4b | D;V | Specify the key study dates, including start of accrual; end of accrual; and, if applicable, end of follow-up. | 8 |
| Participants | 5a | D;V | Specify key elements of the study setting (e.g., primary care, secondary care, general population) including number and location of centres. | 7 |
|  | 5b | D;V | Describe eligibility criteria for participants. | 7 |
|  | 5c | D;V | Give details of treatments received, if relevant. | 7 |
| Outcome | 6a | D;V | Clearly define the outcome that is predicted by the prediction model, including how and when assessed. | 7-9 |
|  | 6b | D;V | Report any actions to blind assessment of the outcome to be predicted. | NA |
| Predictors | 7a | D;V | Clearly define all predictors used in developing or validating the multivariable prediction model, including how and when they were measured. | 7-8 |
|  | 7b | D;V | Report any actions to blind assessment of predictors for the outcome and other predictors. | NA |
| Sample size | 8 | D;V | Explain how the study size was arrived at. | 7 |
| Missing data | 9 | D;V | Describe how missing data were handled (e.g., complete-case analysis, single imputation, multiple imputation) with details of any imputation method. | 7 |
| Statistical analysis methods | 10a | D | Describe how predictors were handled in the analyses. | 7 |
|  | 10b | D | Specify type of model, all model-building procedures (including any predictor selection), and method for internal validation. | 9-10 |
|  | 10c | V | For validation, describe how the predictions were calculated. | 8-10 |
|  | 10d | D;V | Specify all measures used to assess model performance and, if relevant, to compare multiple models. | 8-9 |
|  | 10e | V | Describe any model updating (e.g., recalibration) arising from the validation, if done. | NA |
| Risk groups | 11 | D;V | Provide details on how risk groups were created, if done. | NA |
| Development vs. validation | 12 | V | For validation, identify any differences from the development data in setting, eligibility criteria, outcome, and predictors. | 8 |
| **Results** | | | | |
| Participants | 13a | D;V | Describe the flow of participants through the study, including the number of participants with and without the outcome and, if applicable, a summary of the follow-up time. A diagram may be helpful. | Figure 1 |
|  | 13b | D;V | Describe the characteristics of the participants (basic demographics, clinical features, available predictors), including the number of participants with missing data for predictors and outcome. | 11,  Table 1 |
|  | 13c | V | For validation, show a comparison with the development data of the distribution of important variables (demographics, predictors and outcome). | 11-12  Figure 3,  Figure S2,  Table 1 |
| Model development | 14a | D | Specify the number of participants and outcome events in each analysis. | 11  Table 1 |
|  | 14b | D | If done, report the unadjusted association between each candidate predictor and outcome. | NA |
| Model specification | 15a | D | Present the full prediction model to allow predictions for individuals (i.e., all regression coefficients, and model intercept or baseline survival at a given time point). | 12 |
|  | 15b | D | Explain how to use the prediction model. | 12  Figure S1 |
| Model performance | 16 | D;V | Report performance measures (with CIs) for the prediction model. | 11-12,  Figure 3 |
| Model-updating | 17 | V | If done, report the results from any model updating (i.e., model specification, model performance). | NA |
| **Discussion** | | | | |
| Limitations | 18 | D;V | Discuss any limitations of the study (such as nonrepresentative sample, few events per predictor, missing data). | 16 |
| Interpretation | 19a | V | For validation, discuss the results with reference to performance in the development data, and any other validation data. | 11-12 |
|  | 19b | D;V | Give an overall interpretation of the results, considering objectives, limitations, results from similar studies, and other relevant evidence. | 14-16 |
| Implications | 20 | D;V | Discuss the potential clinical use of the model and implications for future research. | 16-17 |
| **Other information** | | | | |
| Supplementary information | 21 | D;V | Provide information about the availability of supplementary resources, such as study protocol, Web calculator, and data sets. | 12-13 |
| Funding | 22 | D;V | Give the source of funding and the role of the funders for the present study. | 19 |

**Table S1**. Transparent reporting of a multivariable prediction model for individual prognosis or diagnosis (TRIPOD) statement

*Items relevant only to the development of a prediction model are denoted by D, items relating solely to a validation of a prediction model are denoted by V, and items relating to both are denoted D;V. We recommend using the TRIPOD Checklist in conjunction with the TRIPOD Explanation and Elaboration
